# Anti‐cancer effect of targeting fibroblast activation protein alpha in glioblastoma through remodeling macrophage phenotype and suppressing tumor progression

**DOI:** 10.1111/cns.14024

**Published:** 2022-11-15

**Authors:** Yazhou Miao, Yuxuan Deng, Jinqiu Liu, Jing Wang, Boyi Hu, Shuyu Hao, Herui Wang, Zhe Zhang, Zeping Jin, Yang Zhang, Chunzhao Li, Peng Zhang, Hong Wan, Shaodong Zhang, Jie Feng, Nan Ji

**Affiliations:** ^1^ Department of Neurosurgery, Beijing Tiantan Hospital Capital Medical University Beijing China; ^2^ National Clinical Research Center for Neurological Diseases (China) Beijing China; ^3^ Beijing Neurosurgical Institute Capital Medical University Fengtai, Beijing China; ^4^ Neuro‐Oncology Branch, Center for Cancer Research National Cancer Institute, National Institutes of Health Bethesda Maryland USA; ^5^ Beijing Cancer Institute, Beijing Institute for Brain Disorders Capital Medical University Beijing China; ^6^ Beijing Advanced Innovation Center for Big Data‐Based Precision Medicine, School of Engineering Medicine Beihang University Beijing China

**Keywords:** epithelial–mesenchymal transition, fibroblast activation protein alpha, glioblastoma, PT100

## Abstract

**Introduction:**

Glioblastoma (GBM) is the most malignant form of glioma and has a poor median survival time. Fibroblast activation protein alpha (FAP) is a dual‐specificity serine protease that is strongly associated with the development and progression of human carcinomas. However, relatively little is known about the function of FAP and its potential as a therapeutic target in GBMs.

**Aims:**

In this study, we aimed to explore the role of FAP in GBM through a series of experiments and to evaluate the therapeutic effect of PT100, a small molecule inhibitor of FAP, on GBM.

**Results:**

Increased FAP expression was associated with poor survival in glioma. In vitro, FAP knockdown inhibited the process of EMT and caused a decrease in the number of M2 macrophages. In vivo, PT100 was confirmed to suppress the progression of GBMs significantly.

**Conclusions:**

FAP could serve as a biomarker and novel therapeutic target for the treatment of GBM and that PT100 is a promising drug for the treatment of GBM.

## Graphical abstract

Fibroblast activation protein alpha (FAP) promotes the progression of glioblastoma by remodeling the morphology of tumor cells and the phenotype of macrophages. PT100, a small molecule inhibitor of FAP, is a promising drug for glioblastoma.

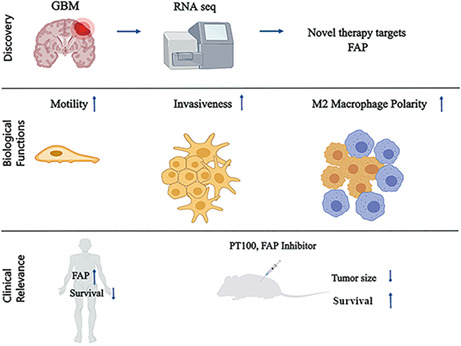

## 1 INTRODUCTION

Gliomas are the most common primary intracranial tumors and can be classified into LGGs and GBM.^1^ GBM is the most malignant form of glioma and has a poor median survival time.^2^, ^3^ The current treatment methods for GBM mainly include surgery, radiotherapy, and chemotherapy. Due to the highly invasive nature of GBMs, it is difficult to achieve complete resection through surgery. Moreover, most GBMs are resistant to radiotherapy and chemotherapy. Thus, there is an urgent need to develop effective therapeutic strategies for treating GBM.

Fibroblast activation protein alpha (FAP) is a homodimeric integral membrane gelatinase belonging to the serine protease family. FAP has become a therapeutic target for epithelial tumors due to its specific expression in epithelial tumors but not in normal tissues. Various therapeutic approaches targeting FAP, such as low‐molecular‐weight inhibitors, antibodies,^4^, ^5^ vaccines,^6^, ^7^ and chimeric antigen receptor (CAR) T cells,^8^, ^9^ have been increasingly developed. A small molecular inhibitor of FAP, PT100 (Val‐boro‐Pro, talabostat), which competitively inhibits the dipeptidyl peptidase activity of FAP, inhibits myeloma tumor growth and bone disease in myelomatous severe combined immunodeficient‐hu mice.^10^ Similarly, oral administration of PT100 to mice slowed the growth of tumors derived from fibrosarcoma, lymphoma, melanoma, and mastocytoma cell lines but did not affect tumor cell growth or viability in vitro.^11^ The mechanism probably involves the production of cytokines and chemokines capable of promoting antitumor immune responses. PT100 has been shown to improve the response to chemotherapy in a colon cancer model. PT100 combined with oxaliplatin significantly inhibited tumor growth and prolonged survival compared to monotherapy, as the dual‐treatment group had fewer M2 macrophages and decreased vascularity.^12^ However, it is not clear whether it can be used as a potent target for the treatment of GBM.

In a word, in this study, we analyzed the function of FAP and its potential as a therapeutic target in GBMs. A series of experimental models were established to investigate the effects of FAP on immune suppression and tumor progression in GBM therapy in vitro and in vivo, and PT100, a small molecule inhibitor of FAP, was also evaluated for the treatment of GBM.

## 2 MATERIALS AND METHODS

### 2.1 Clinical samples

GBM (WHO IV, *n* = 23) and LGG (WHO II, *n* = 17) surgical specimens were obtained for RNA sequencing. A total of 17 samples were obtained for the tissue microarray (TMA), including 28 WHO grade II glioma samples, 31 WHO grade III glioma samples, and 113 GBM samples. In this study, all the samples were obtained from Beijing Tiantan Hospital from June 2014 to September 2019. The study was approved by the Research Ethics Committee of Beijing Tiantan Hospital (KY2014‐021‐02), and informed consent was obtained from all the patients.

### 2.2 RNA sequencing and gene set enrichment analysis (GSEA)

RNA was isolated from the tumors with TRIzol reagent (Invitrogen). Sequencing libraries were generated using rRNA‐depleted RNA using a NEBNext Ultra Directional RNA Library Prep Kit for Illumina (NEB) following the manufacturer's recommendations. Finally, Illumina HiSeq2000 was used to sequence libraries, and transcripts with an adjusted *p* < 0.05 were considered to be differentially expressed.

### 2.3 RNA sequencing and clinical information from TCGA data repository

We downloaded clinical and RNA sequencing data from TCGA. A total of 152 glioblastoma samples, 216 WHO grade II glioma samples, and 241 WHO grade III glioma samples were used for our study excluding samples from patients with incomplete clinical information. Gene expression Profiling Interactive Analysis 2 (http://gepia2.cancer‐pku.cn) was used to determine the effect of FAP on the prognosis of LGG and GBM patients.

### 2.4 Immunohistochemical staining and quantification

IHC staining was performed using a Leica BOND III automated system. Anti‐FAP antibody (ab207178, 1:400) was used for the IHC staining of tumor tissues from patients with glioma. Immunostained slides of TMA sections were scanned using a Leica Aperio AT2 scanner (at 400× magnification) and analyzed using a Leica Aperio ImageScope v12.3. The automated algorithm scored the staining of each tissue core as negative (0), weak (1+), moderate (2+), or strong (3+) according to the scoring criteria threshold. The algorithms also determined the percentage of positive staining. Then, the H‐score of each case was established using the formula H‐score = 1 × (percentage of 1+ cores) + 2 × (percentage of 2+ cores) + 3 × (percentage of 3+ cores). Thus, the H‐score ranged from 0 to 300.^13^ FAP (ab207178), Ki‐67 (ab15580), E‐cad (Cell Signaling Technology, #14472), and N‐cad (Cell Signaling Technology, #13116) antibodies were used for the IHC staining of subcutaneous tumors from BALB/c athymic nude mice.

### 2.5 Cell culture

The human glioma cell lines LN18 and LN229 and the human monocytic cell line U937 were purchased from the American Type Culture Collection. LN18 and LN229 cells were cultured in DMEM supplemented with 10% fetal bovine serum (FBS) and 1% penicillin–streptomycin at 37°C with 5% CO_2_. U937 cells were cultured in RPMI‐1640 supplemented with 10% FBS and 1% penicillin–streptomycin at 37°C with 5% CO_2_. The cell lines used in this study tested negative for mycoplasma using the TransDetect PCR Mycoplasma Detection Kit (TransGen).

### 2.6 Lentiviral infection

Lentiviral plasmids carrying FAP or shRNA targeting FAP were used to upregulate or knock down the expression of FAP, respectively, and all lentiviruses were purchased from Vigene Biosciences, Inc.

### 2.7 Immunostaining and fluorescence microscopy

Approximately 2 × 10^4^ cells were plated in each well of a 24‐well plate containing glass coverslips. After 12 h of incubation, the cells were fixed with 4% paraformaldehyde, permeabilized with 0.1% Triton X‐100, blocked with goat serum, incubated in FAP antibodies (Bioworld Technology, Inc., BS90492), and incubated in secondary antibodies. Nuclear DNA was stained with 4,6‐diamidino‐2‐phenylindole (DAPI) at a 1:1000 dilution. F‐actin was stained with Alexa Fluor 488‐conjugated phalloidin (Invitrogen, A12379). The fluorescently labeled cell samples were visualized with a Zeiss Axio Observer. Z1 epifluorescence microscope (Zeiss).

### 2.8 Scanning electron microscopy (SEM) sample preparation and observation

A total of 2 × 10^4^ cells were plated in each well of a 24‐well plate containing glass coverslips. After the cells adhered to the glass coverslips, the cell slides were washed with phosphate‐buffered saline (PBS), collected into an Eppendorf tube, and fixed in 4% paraformaldehyde and 2.5% glutaraldehyde at 4°C for 2 h. Then, the cells were dehydrated, displaced, dried, and sprayed. Images were acquired with a Hitachi TM‐1000 (Hitachi) scanning electron microscope.

### 2.9 Cell invasion and migration assays

Corning Matrigel Invasion Chamber with 8.0 μm polyethylene terephthalate Membrane (CORNING, 354480) was used to conduct cell invasion assays. And Transwell Permeable Supports with 6.5 μm Polycarbonate Membrane (CORNING, 3421) were performed to assay cell migration. DMEM without FBS was used to resuspend the cells and placed in the upper chamber wells, whereas DMEM with 10% FBS was added to the lower chamber as the chemoattractant. After 24 h for invasion assay and 12 h for migration assay, the cells were washed with PBS solution, fixed with 4% paraformaldehyde, and stained with 0.1% crystal violet solution. The numbers of migrated and invaded cells were counted under a microscope. Cell imaging was done using an inverted microscope and ImageJ software was used to quantify the cells. All assays were performed in triplicate.

### 2.10 Wound healing assay

Equal amounts of cells were plated and allowed to grow to 90% confluence. Culture inserts for live cell analysis (Ibidi) were used to make a wound in the cell monolayer. The wound areas were marked and photographed at 0 and 24 h with a phase‐contrast microscope. ImageJ software was used to calculate the area of wound healing. All assays were performed in triplicate.

### 2.11 Reverse transcription‐quantitative polymerase chain reaction (RT‐qPCR)

Total RNA was isolated from glioma cell lines using QIAzol Lysis Reagent (Qiagen Sciences). The cDNA was synthesized using a High Capacity cDNA Reverse Transcription Kit (Applied Biosystems) and analyzed using QuantStudio 5 (Applied Biosystems). The amplification program was as follows: initial denaturation step at 95°C for 30 s, followed by 40 cycles at 95°C for 15 s and 60°C for 60 s. The primer sequences are shown in Table S1. All assays were performed in triplicate.

### 2.12 Western blotting analysis

The LN18 and LN229 cells were harvested with 0.05% trypsin, washed with cold PBS, and lysed with ice‐cold lysis buffer supplemented with protease inhibitors. The proteins were resolved using gel electrophoresis with 10% Tris‐glycine gels and transferred onto polyvinylidene fluoride (PVDF) membranes. After blocking the nonspecific binding sites with 5% nonfat milk in TBST buffer, the membranes were incubated with primary antibodies. The membranes were then incubated with the appropriate peroxidase‐conjugated secondary antibodies, and the specific protein bands were visualized using enhanced chemiluminescence reagents. In this study, epithelial phenotype‐related proteins, mesenchymal phenotype‐related proteins, and transcription factors associated with EMT, such as E‐cadherin, N‐cadherin, Snai1, and Twist, were tested to observe the process of EMT. The antibodies used in this study were as follows: anti‐FAP primary antibody was purchased from Bioworld Technology, Inc. (BS90492); anti‐Twist (ab50581) and anti‐β‐actin (ab8227) primary antibodies were purchased from Abcam; and anti‐N‐cadherin, anti‐E‐cadherin, and anti‐Snail primary antibodies were purchased from Cell Signaling Technology with catalog numbers of #13116, #14472, and #3879, respectively. All assays were performed in triplicate.

### 2.13 Macrophage polarization

U937 cells were cultured in the suggested medium for 24 h before priming. U937 monocytes were primed with 80 ng/ml phorbol 12‐myristate‐13‐acetate (PMA) (Abcam, ab120297) for 48 h to induce the formation of monocyte‐derived macrophages.^14^ Then, flow cytometry was used to verify whether U937 cells were polarized into macrophages by detecting the expression of CD68 (BioLegend, 564944) and CD11b (BioLegend, 301345), the markers of macrophages. All assays were performed in triplicate.

### 2.14 Flow cytometric analysis

For U937‐derived macrophages, conditioned medium from LN229 cells or FAP‐knockdown LN229 cells was added and incubated for 48 h. After incubation at 37°C for 48 h, the cells were harvested with 0.05% trypsin, washed with PBS, fixed with FluoroFix Buffer (BioLegend, 422101), incubated with anti‐CD206 (BioLegend, 321106) and anti‐CD163 (BioLegend, 326514) or isotype control antibodies at room temperature. The cells were centrifuged and washed with FACS buffer. The samples were analyzed in an Amnis ImageStreamX MarkII (Luminex). For data analysis, FlowJo software (Becton Dickinson) and IDEAS 6.2 were used. The expression of CXCL8 in LN229 cells with or without FAP expression knocked down was measured by flow cytometry as described above. All assays were performed in triplicate.

### 2.15 Subcutaneous tumor formation and intracranial tumor formation

Six‐week‐old female BALB/c athymic nude mice and C57BL/6 mice were purchased from Charles River Laboratories. All experiments were approved by Beijing Neurosurgical Institute following the U.K. Animals (Scientific Procedures) Act (1986) and its associated guidelines. For BALB/c athymic nude mice, a total of 5 × 10^6^ viable LN229 cells were transplanted into the right shoulders. The tumor volume was measured every 3 days and calculated with the following formula: *V* = *a* × *b* ^2^ × 0.5 (*a* is the longest diameter and *b* is the diameter perpendicular to *a*). At the end of the experiment, the tumors were removed, weighed, photographed, and subjected to hematoxylin and eosin (H&E) staining and immunohistochemical analysis. For C57BL/6 mice, GL261 cells (200,000 cells in 5 μl PBS) expressing luciferase by lentiviral infection (GL261‐luc) were slowly injected into the brain on the coronal suture 2.0 mm lateral (right) to bregma, at a depth of 3.0 mm using a Hamilton syringe. Bone wax was used to close the hole, and the wound was sutured. Twenty days after the surgery, the C57BL/6 mice were imaged using the In Vivo Imaging System (IVIS, PerkinElmer) 10 min after 150 mg/kg D‐luciferin (PerkinElmer, 122799) was intraperitoneally injected. In tumor bioluminescence images, fluorescence intensity could reflect the volume of the tumor. Fluorescence intensity was quantified using Living Image, a software program provided by the same manufacturer. The mice were imaged every 7 days using the IVIS system to evaluate tumor volume. The mice were euthanized when they exhibited neurological signs, including hydrocephalus and hemiparesis. The investigators were not blinded to allocation during experiments and outcome assessment. All the animal protocols were approved by the Animal Welfare Ethics Committee of Beijing Neurosurgical Institute.

### 2.16 Statistical analysis

R (version 4.0.4, http://www.r‐project.org) was used for statistical analysis and visualization in this study. Several R packages, such as ggplot2, ggpubr, and survival ROC, were used to generate figures. All the quantitative data presented are the mean ± SEM from at least three samples or experiments per data point, and the statistical values were calculated with GraphPad Prism 8.0 (GraphPad Software, Inc.). The normality of the data distribution was analyzed by the Shapiro–Wilk test. Differences between the two groups were evaluated with Student's *t*‐test or Mann–Whitney *U* (nonparametric tests), and differences among the three groups were evaluated using one‐way ANOVA or Kruskal–Wallis test (nonparametric tests). *p* < 0.05 was considered to indicate a significant difference.

## 3 RESULTS

### 3.1 The key EMT‐related gene FAP is highly expressed in GBM according to transcriptomics

**FIGURE 1:**
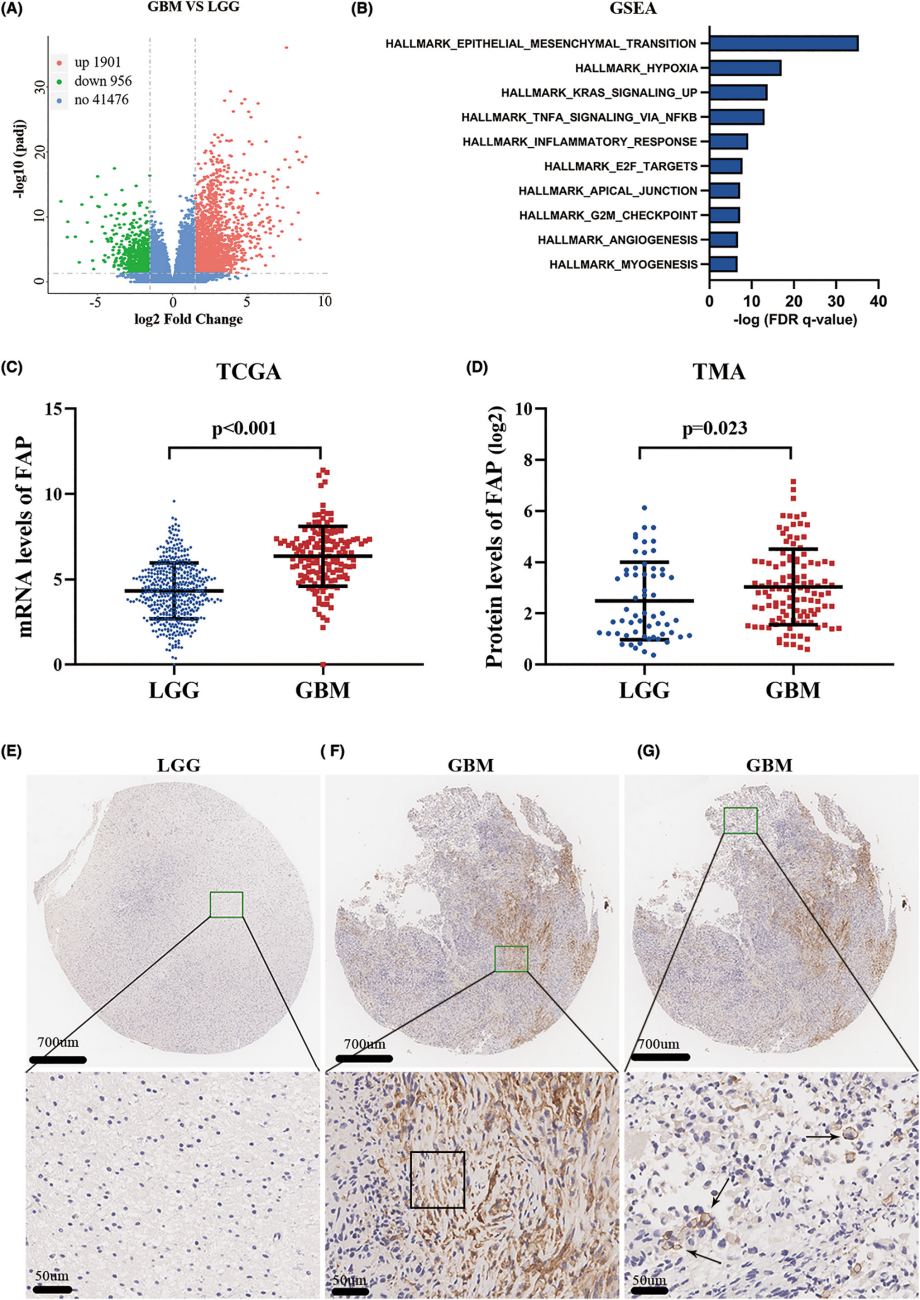
Fibroblast activation protein alpha (FAP) is highly expressed in GBM. (A) Volcano plots of the differentially expressed genes (DEGs) in GBM and LGG. The red dot represents the genes with adjusted *p* < 0.01 and log_2_FC >1, and the green dot represents the genes with adjusted *p* < 0.01 and log_2_FC < −1. (B) The top 10 enriched gene sets of the differentially expressed genes between LGG and GBM were determined based on the molecular signature database in GSEA. (C, D) FAP expression was significantly increased in GBM compared with LGG in TCGA (C) and TMA datasets (D). (E–G) Typical immunohistochemistry image of FAP expression in LGG (E) and GBM (F, G), FAP was expressed in the extracellular matrix (F) and cytomembrane (G). Rectangles represent the expression of FAP in the extracellular matrix (F) and the arrows represent the expression of FAP in the cell membrane(G).

In this study, mRNA expression profiling was used to identify differentially expressed genes between 23 GBM samples and 17 WHO grade II glioma samples. There were 2235 upregulated genes and 1217 downregulated genes in GBM compared to WHO grade II glioma based on the cutoff criteria of |log_2_fold change (FC)| > 1.0 and adjusted *p*‐value<0.01 (Figure 1A). These differentially expressed genes were enriched in different signaling pathways according to GSEA. The EPITHELIAL_MESENCHYMAL_TRANSITION pathway is the top pathway and could play an important role in GBM (Figure 1B). Among the genes related to the EMT pathway, FAP was more highly expressed in GBM samples than in WHO grade II glioma samples (*p* < 0.05, Student's *t*‐test). Furthermore, FAP expression was significantly upregulated in GBM compared to LGG according to TCGA data (Figure 1C, *p* < 0.05, Student's *t*‐test). Moreover, IHC was used to validate the aberrant expression of FAP. Consistent with the above results, IHC showed that FAP was frequently overexpressed in GBM samples (Figure 1D–G) compared to LGG samples. These data demonstrated that FAP was preferentially expressed in GBMs.

### 3.2 Elevated FAP expression in glioma patients with wild‐type IDH1, non‐codeletion of 1p19q, old age, and poor prognosis

**FIGURE 2:**
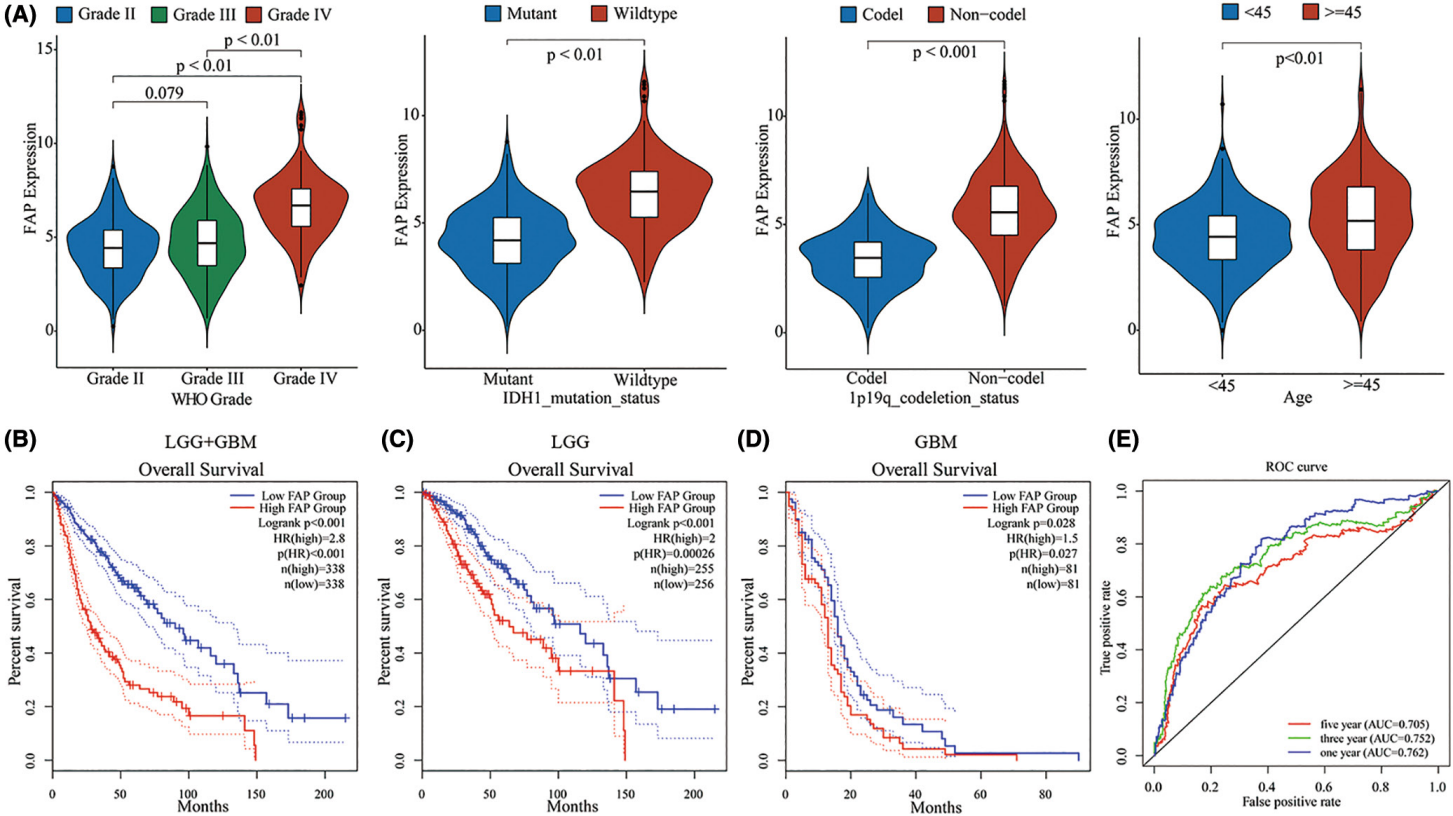
High fibroblast activation protein alpha (FAP) expression indicated a poorer prognosis in glioma patients. (A) Clinical features are significantly associated with the expression of FAP in TCGA databases. (B, C, D) Survival analysis of FAP in glioma patients in TGGA dataset. Patients with high expression of FAP had poorer survival in the LGG (C) and GBM (D). (E) ROC curve showing the predictive value of FAP expression for glioma in TCGA databases.

The correlation analysis indicated that FAP expression was related to 1p19q codeletion status, IDH1 mutation status, and age. High FAP expression was often found in patients with non‐codeletion of 1p19q, wild‐type IDH1 status, and old age (Figure 2A). We further evaluated the relationship between FAP expression level and overall survival. Patients with gliomas in the TCGA cohort were divided into two groups according to the expression level of FAP. The group with a high expression of FAP had a poorer prognosis in whole grades (Figure 2B, *p* < 0.001, log‐rank test). We also investigated the relationship between the expression of FAP and the overall survival of patients in the LGG and GBM cohorts and found that patients with high expression of FAP had poorer survival in both cohorts (Figure 2C,D, *p* < 0.05, log‐rank test). Then, we used FAP expression and overall survival data to generate receiver operating characteristic (ROC) curves based on the TCGA database. The area under the curve (AUC) was 0.762 when predicting 1‐year survival, 0.752 when predicting 3‐year survival, and 0.705 when predicting 5‐year survival (Figure 2E). In conclusion, FAP predicts a poor prognosis for glioma patients and might serve as a biomarker for predicting overall survival.

### 3.3 FAP regulates EMT and promotes GBM invasion

**FIGURE 3:**
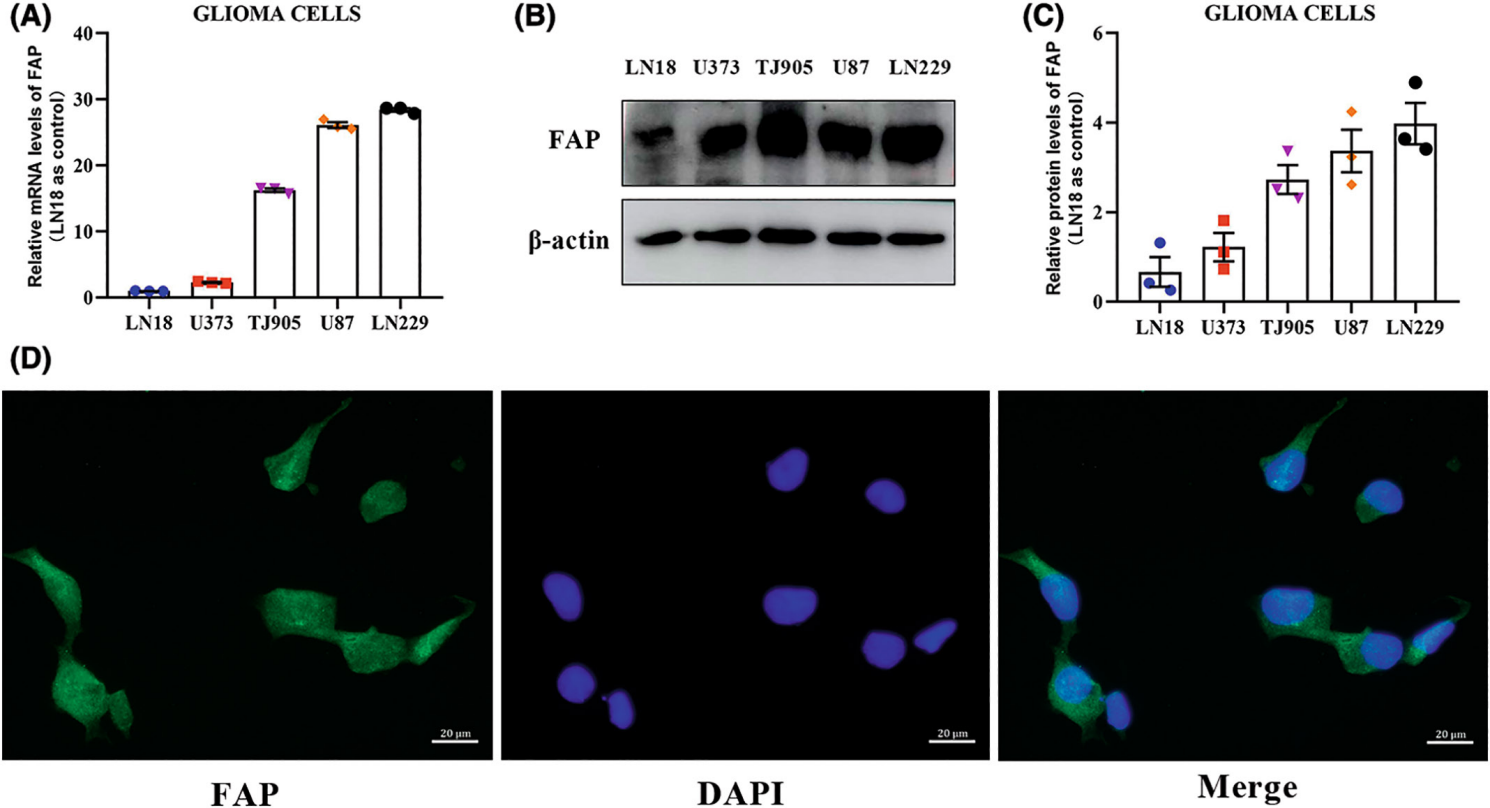
Fibroblast activation protein alpha (FAP) is expressed in the cytoplasm and nucleus in glioblastoma cells. (A) RT‐PCR was used to evaluate the mRNA levels of endogenous FAP in various glioma cell lines. The mRNA level of FAP in LN18 cells was used as a control. (B) Western blotting analysis of the protein levels of endogenous FAP in various glioma cell lines. (C) The quantification of the protein levels of endogenous FAP in various glioma cell lines. The protein level of FAP in LN18 cells was used as a control. (D) The localization of FAP was detected by immunofluorescence assay in LN229 cells. Green: FAP, Blue: nucleus.

In this study, RT‐qPCR and western blotting assays were used to evaluate the expression level of FAP in various GBM cell lines, including LN229, U87, U373, TJ905, and LN18 cell lines. According to the results, the expression of FAP was highest in LN229 cells and lowest in LN18 cells (Figure 3A–C). Then, the localization of FAP in LN229 cells was detected by immunofluorescence assay. Immunofluorescence assays showed that FAP was expressed in both the cytoplasm and nucleus of LN229 cells (Figure 3D). Moreover, the IHC of tumor tissues showed that FAP was also expressed in the extracellular matrix (Figure 1F) and cytomembrane (Figure 1G).

**FIGURE 4:**
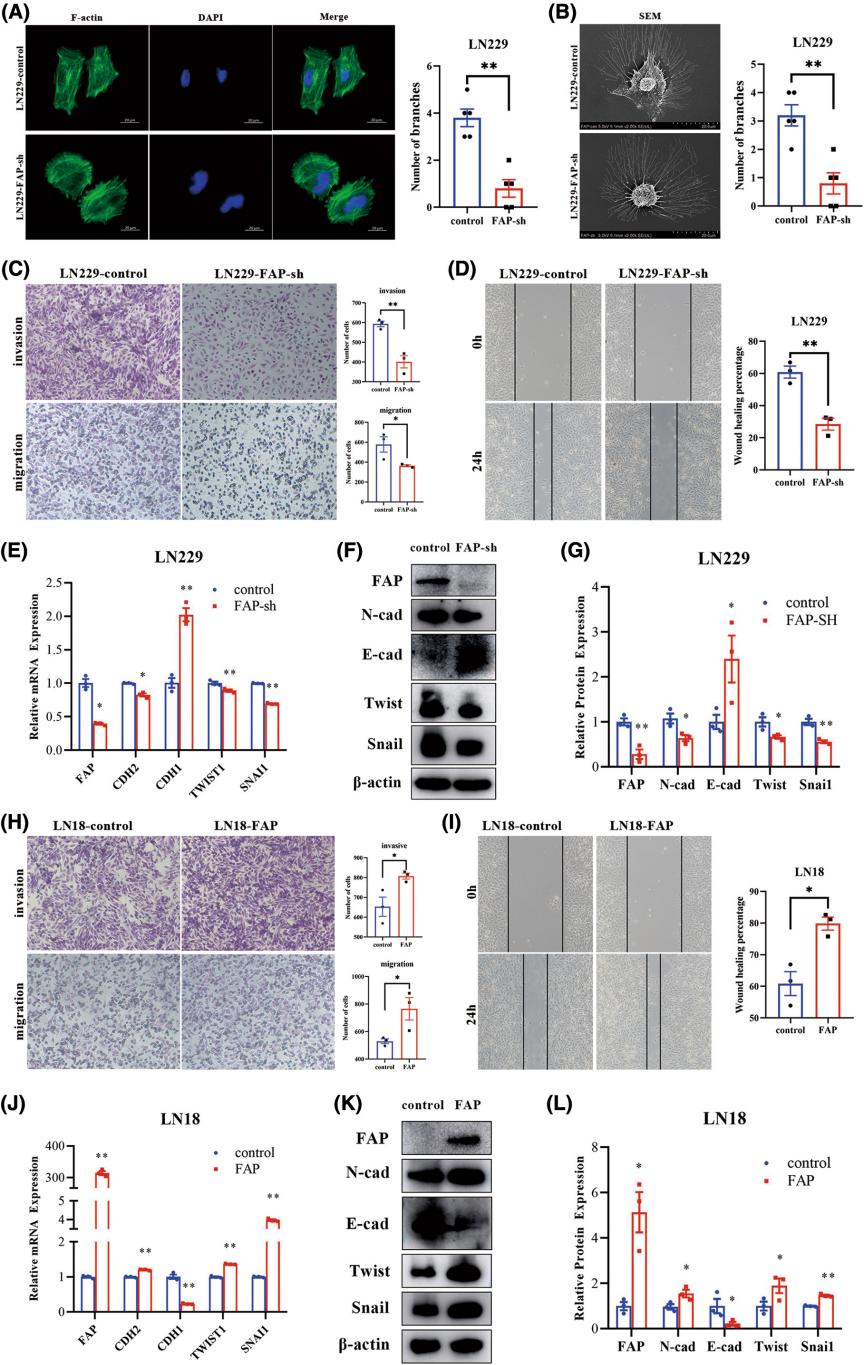
Fibroblast activation protein alpha (FAP) could facilitate the invasion and migration of GBM cells and promote EMT in GBM cells. (A) Representative images of the cytoskeleton showed that FAP affected cellular morphology and the morphological change was quantified using the number of branches. (B) Representative SEM images showed that FAP affected cellular morphology and the morphological change was quantified using the number of branches. (C) Transwell assays were used to detect the invasive and migratory capacities of LN229 cells. (D) A wound‐healing assay was used to detect the migratory capacities of LN229 cells. (E) RT‐PCR analysis of FAP and EMT markers expression in LN229. (F) Western blotting analysis of FAP and EMT markers expression in LN229 cells. (G) The relative protein expression level normalized to the β‐actin protein expression level in LN229 cells. (H) Transwell assays were used to detect the invasive and migratory capacities of LN18 cells. (I) A wound‐healing assay was used to detect the migratory capacities of LN18 cells. (J) RT‐PCR analysis of FAP and EMT markers expression in LN18. (K) Western blotting analysis of FAP and EMT markers expression in LN18 cells. (L) The relative protein expression level normalized to the β‐actin protein expression levels in LN18 cells. *p < 0.05. **p < 0.01.

To investigate the function of FAP, shRNA was designed to reduce FAP expression in LN229 cells. When FAP expression was knocked down in LN229 cells, the morphology of LN229 cells changed from a mesenchymal phenotype to an epithelioid phenotype. In this study, F‐actin staining and SEM experiments were used to observe the morphological changes of LN229 cells, and the cell morphological changes were quantified using the number of branches. LN229 cells without any intervention were polygonal and had more branches, suggesting that the cells had strong deformability and invasion capabilities. The morphology of FAP‐knockdown cells was round, indicating that the deformability of the cells was weakened (Figure 4A,B, *p* < 0.01, Student's *t*‐test). Moreover, FAP knockdown significantly attenuated the invasion and migration of LN229 cells, according to transwell and wound healing assay results (Figure 4C,D, *p* < 0.01, Student's *t*‐test). Epithelial–mesenchymal transition is a process in which epithelial cells lose their polarity and increase their migration and motility, while the epithelial phenotype is lost and the mesenchymal phenotype is gradually acquired. Along with the phenotypic changes, the expression of many proteins is altered, with a decrease in epithelial phenotype‐related proteins, such as E‐cadherin, and an increase in mesenchymal phenotype‐related proteins, such as N‐cadherin, as well as changes in the expression of a series of transcription factors such as Snai1 and Twist. Therefore, RT‐qPCR and western blotting assays were used to detect changes in E‐cadherin, N‐cadherin, Snai1, and Twist expression after FAP knockdown in LN229 cells. According to the results, the level of the epithelial marker E‐cadherin was enhanced, and the levels of the mesenchymal markers N‐cadherin, Snai1, and Twist were decreased when FAP expression was knocked down (Figure 4E–G, *p* < 0.05, Student's *t*‐test). In addition, stable FAP‐overexpressing cells were constructed with the LN18 cell line, which has low endogenous FAP expression. In contrast to LN229 cell lines with FAP expression knocked down, LN18 cell lines overexpressing FAP exhibited enhanced invasion and migration in transwell and wound healing assays (Figure 4H,I, *p* < 0.05, Student's *t*‐test). Moreover, the overexpression of FAP decreased the expression of E‐cadherin and increased the expression of N‐cadherin, Snai1, and Twist in LN18 cells (Figure 4J–L, *p* < 0.05, Student's *t*‐test). Together, these results indicated that FAP could facilitate the invasion and migration of GBM cells and promote the EMT of GBM cells.

### 3.4 FAP induces CXCL8 expression to increase M2 macrophage polarization

**FIGURE 5:**
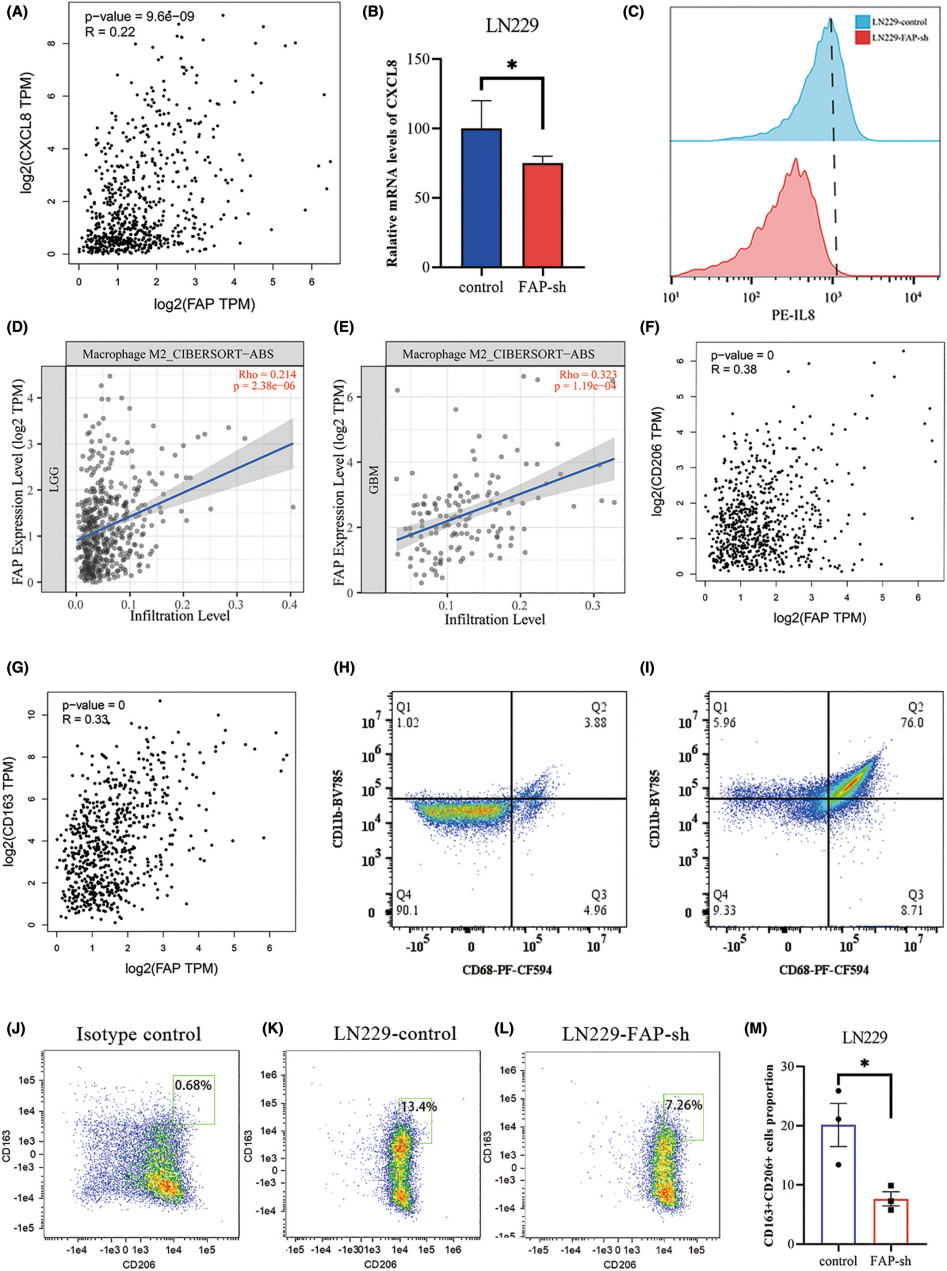
Fibroblast activation protein alpha (FAP) induces CXCL8 expression to increase M2 macrophage polarization. (A) Correlation between the expression levels of FAP and CXCL8 in TCGA databases. (B) RT‐PCR analysis of CXCL8 expression in LN229 cell lines after FAP knockdown. (C) Flow cytometry analysis of CXCL8 expression in LN229 cell lines after FAP knockdown. (D and E) TIMER2.0 prediction results showed that FAP expression was positively correlated with M2‐type macrophage numbers in LGG (D) and GBM (E). (F and G) The mRNA expression of FAP was positively correlated with the expression of the M2‐type macrophage markers CD206 (F) and CD163 (G) in TCGA databases. (H and I) U937 cells were induced to differentiate into M0‐type macrophages under the action of PMA. Flow cytometry was used to detect the expression of M0‐type macrophage markers CD68 and CD11b, and the population of CD68+CD11b+ cells was significantly increased in PMA‐induced U937 cells (I) compared with U937 cells not induced with PMA (H). (J–M) FAP in GBM cells could increase M2‐type macrophage polarization. Flow cytometry was used to detect the expression of CD163 and CD206 in U937‐derived macrophages after 48 h of culture with the supernatant of LN229 cells from the control group (K) and FAP‐knockdown group (L), and the population of CD163+CD206+ cells was significantly increased in the control group (M). *p < 0.05.

Based on the TCGA dataset, we found a significant positive correlation between the expression of FAP and CXCL8 (Figure 5A, *p* < 0.001, Pearson's *r* test). Furthermore, the expression of CXCL8 was shown to be significantly decreased after FAP expression was knocked down according to RT‐qPCR and flow cytometry (Figure 5B,C, *p* < 0.05, Student's *t*‐test). Previous studies have shown that CXCL8 can induce macrophage polarization toward the M2 type.^15^, ^16^ In this study, the Tumor IMmune Estimation Resource (TIMER) was used to predict the correlation between FAP expression and M2‐type macrophage numbers in LGG and GBM. The results showed that there was a strong positive correlation between the expression of FAP and the infiltration level of M2‐type macrophages in both LGG and GBM (Figure 5D,E, *p* < 0.001, Pearson's *r* test). The expression of FAP was also strongly positively correlated with the expression of CD206 and CD163, markers of M2‐type macrophages, based on the TCGA dataset (Figure 5F,G, *p* < 0.001, Pearson's *r* test). In previous studies, CD68, CD11b, and CD14 are often used to define M0‐type macrophages, while CD206, CD163, and ARG1 are often used to define M2‐type macrophages.^17^, ^18^, ^19^ To better define the cell population, in this study, we used CD68 and CD11b to define M0 macrophages, and CD206 and CD163 to define M2‐type macrophages. The results of flow cytometry showed that the expression levels of CD68 and CD11b in U937 monocytes were significantly increased under the induction of PMA. This result demonstrated that U937 monocytes could be induced to differentiate into monocyte‐derived macrophages, also called M0‐type macrophages under the stimulation of PMA (Figure 5H,I). U937‐derived macrophages were cultured for 48 h with the conditioned medium of LN229 cells with or without FAP expression knocked down. Flow cytometry was used to detect the expression of CD206 and CD163 in U937‐derived macrophages. The results showed that the expression of CD206 and CD163 in U937‐derived macrophages cultured in the conditioned medium from FAP‐knockdown cells was significantly reduced (Figure 5J–M, *p* < 0.05, Student's *t*‐test), indicating that FAP knockdown induces a decrease in the M2 polarization of macrophages. Taken together, FAP in GBM cells could affect the M2 polarization of macrophages by potentially regulating the expression of CXCL8.

### 3.5 PT100 is a potent antitumor agent for GBM treatment in vivo

**FIGURE 6:**
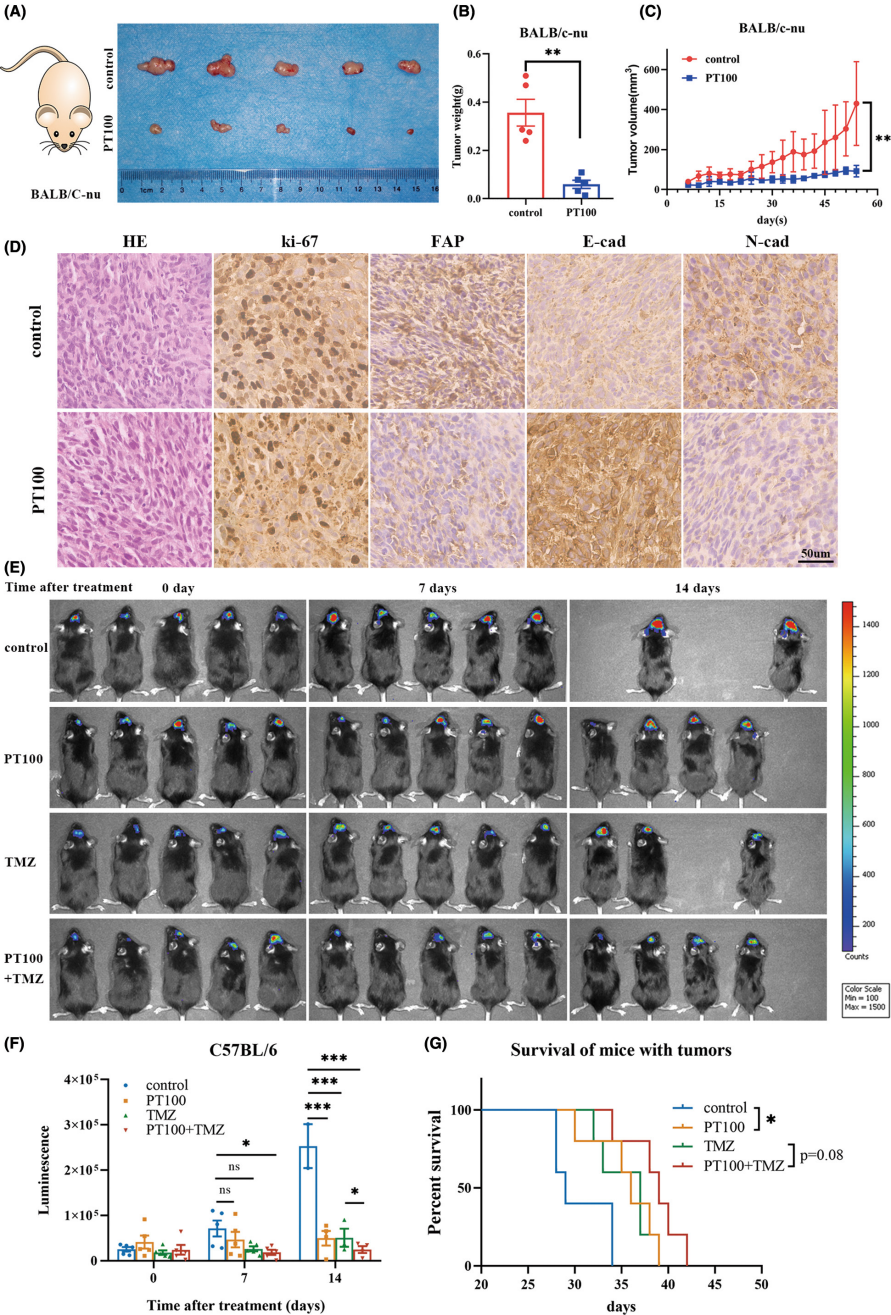
PT100 exhibits a remarkable antitumor effect in vivo. (A) Macroscopic image of resected tumors of subcutaneous xenograft model in BALB/c nude mice. (B) Histogram of tumor weight (*n* = 5) of subcutaneous xenograft model in BALB/c nude mice. (C) Tumor volume of BALB/c nude mice treated with PT100 every 3 days (*n* = 5). (D) Representative images of HE and immunohistochemical staining for Ki‐67, FAP, E‐Cadherin, and N‐Cadherin in the tumors of BALB/c nude mice. Bar = 50 μm. (E) Tumor bioluminescence images of C57BL/6 mice at 0, 7, and 14 days after treatment with PT100, TMZ, or their combination in an orthotopic allograft model generated with GL261 cells (*n* = 5). The missing mice in the graph were death at 14 days after treatment. (F) The bioluminescence intensity of mice at 0, 7, and 14 days after treatment. (G) Kaplan–Meier survival curve of C57BL/6 mice with GBM (*n* = 5).

PT100 is an orally active and nonselective inhibitor of FAP.^11^ Here, we examined the efficacy of PT100 in the treatment of GBM. A BALB/c nude mouse subcutaneous xenograft model and a C57BL/6 intracranial allograft model were used in this study. For the BALB/c nude mice, we randomized the mice into two groups: control and PT100 treatment (2 mg/kg every day). The group of PT100 treatment exhibited smaller tumor volume, lower weight, and slower growth velocity, suggesting that PT100 has powerful antitumor effects in the subcutaneous xenograft model (Figure 6A–C, *p* < 0.001, Student's *t*‐test). Moreover, IHC staining of tumor tissue showed that the expression of Ki‐67, a marker of proliferation, was decreased in the PT100 group, the expression of the EMT‐related marker N‐cadherin was decreased, and the expression of E‐cadherin was increased in the PT100‐treated group of BALB/c nude mice (Figure 6D). Furthermore, GL261‐luc cells were injected intracranially in C57BL/6 mice to construct an intracranial allograft model. At 20 days after GL261‐luc cells were injected, the mice were divided into four groups according to fluorescence intensity to ensure a relatively uniform size of the tumor. The mice in the control group did not receive any treatment, the mice in the PT100 group received 2 mg/kg PT100 (MedChemExpress) every day (oral, p.o.), the mice in the TMZ group received 8 mg/kg TMZ (MedChemExpress) five times per week (intraperitoneal, i.p.), and the mice in the PT100 + TMZ group received 2 mg/kg PT100 every day and 8 mg/kg TMZ five times per week. According to the results, the mice treated with PT100 exhibited smaller tumor sizes and extended survival compared to those in the control group (Figure 6E–G). These results suggested that PT100 has remarkable antitumor effects on GBM.

TMZ, a prodrug alkylating agent, is oral chemotherapy. Studies have shown that adjuvant radiochemotherapy and TMZ provide significant improvements in progression‐free and overall survival rates in the treatment of glioblastoma.^20^, ^21^ To explore whether the combined treatment of PT100 and TMZ is better than treatment with TMZ alone, the TMZ treatment group, and the combined treatment group (PT100 and TMZ) were established in this study. The experimental results showed that the tumor volume of the C57BL/6 mice in the PT100 and TMZ combined treatment group was significantly reduced compared with that in the TMZ treatment group (Figure 6E,F, *p* < 0.05, Student's *t*‐test), and the combined treatment group tended to show a longer survival time (Figure 6G, *p* = 0.08, log‐rank test). Together, these animal studies demonstrate that PT100 is a promising drug for the treatment of GBM.

## 4 DISCUSSION

The restricted expression profile of FAP in normal adult tissues and its overexpression in a variety of pathologies has intensified research efforts focused on the development of anti‐FAP therapies, but the role of FAP in gliomas has been poorly studied. In this study, we first comprehensively analyzed the correlation between the expression of FAP and various clinical features of patients with GBM and found that high expression of FAP suggested a poorer prognosis. Several studies have shown that FAP expression was correlated with worse overall survival across a wide range of human cancers,^22^ including colorectal cancer, clear cell renal cell carcinoma, gastric cancer, intrahepatic cholangiocarcinoma, oral squamous cell carcinoma, ovarian cancer, pancreatic ductal adenocarcinoma, and non‐small cell lung adenocarcinoma.^23^, ^24^, ^25^, ^26^, ^27^, ^28^, ^29^, ^30^, ^31^, ^32^, ^33^, ^34^ In this study, we obtained similar results in LGG and GBM by analyzing the data in TCGA datasets. However, Busek et al. analyzed 56 glioma patients and concluded that the expression level of FAP was not associated with the prognosis of patients, which was inconsistent with our conclusions. The most likely reason for this difference was that their sample size was too small to obtain credible results.^35^ Additionally, we also found that high FAP expression was correlated with non‐codeletion of 1p19q, wild‐type IDH1 status, and old age, which has not been explored in previous studies. Together, according to our analysis, high FAP expression predicts a poorer prognosis for glioma patients, elevated FAP expression correlates with non‐codeletion of 1p19q, wild‐type IDH1 status, and old age, and FAP might serve as a biomarker for predicting overall survival. In previous studies, many proteins have been proposed as prognostic biomarkers for glioblastoma, for example, CXCL1,^36^ TRIB2/MAP3K1,^37^ and RGS16^38^ among others. Compared to the biomarkers mentioned above, FAP is selectively expressed in glioma, and FAP is either not expressed or present at insignificant levels in normal brain tissues. Therefore, FAP is a promising therapeutic target and imaging target for glioma due to its specific expression.

Invasion and migration are the most common features of tumors.^39^, ^40^ Previous studies have shown that FAP has a great influence on tumor invasion and migration. EMT refers to the process by which epithelial cells lose polarity and become migratory mesenchymal cells after cytoskeletal remodeling. EMT makes tumor cells more aggressive and migratory. Based on these results, it is reasonable to speculate that the EMT process was involved in the invasion and migration of glioblastoma cells when FAP was knocked down or overexpressed. In our study, we found that FAP expression had a great impact on the morphology and invasiveness of GBM cells. FAP could promote the EMT process by altering the expression of EMT‐related molecular markers. These results indicated that FAP promotes cell invasion and migration by activating the EMT program, which was consistent with our assumption. Whether FAP affects tumor proliferation is controversial. Some studies suggest that FAP promotes the proliferation of tumor cells. A meta‐analysis of gastric cancer revealed that cases with high FAP expression were enriched in the gene pathways of cell growth.^26^ Gong et al.^41^ also found that tumor cells with more endogenous FAP expression proliferated faster, and treatment with siFAP could reduce this proliferation in gastric cancer. However, Jia et al.^42^ found that there was no significant difference between FAP‐overexpressing cells and control cells of the breast cancer cell line MDA‐MB‐231. In our study, FAP had no significant effect on cell proliferation in vitro regardless of whether the expression of FAP was knocked down or whether the function of FAP was inhibited by its inhibitor PT100 (Figure S1). A plausible reason for this observation may be that FAP functions through different mechanisms in different tumor cells. However, it was noteworthy that PT100 was observed to significantly inhibit tumor growth in vivo in this study. We considered that FAP did not facilitate the proliferation of GBM cells directly, but we speculated that FAP facilitates the proliferation of GBM cells by inducing angiogenesis in vivo according to the previous studies.^43^

Recently published results have shown that M2 macrophages facilitate the progression of GBM, and the high prevalence of M2 macrophages was significantly associated with poorer overall survival, although the exact mechanism remains unclear. In this study, based on the analysis of TCGA data, we found that FAP expression was strongly positively correlated with M2 macrophage numbers in both LGG and GBM. In our research, by detecting the expression of CD206 and CD163, markers of M2 macrophages, we found that high expression of FAP promotes the polarization of U937‐derived macrophages to M2 macrophages. This is the first time that FAP expression was shown to promote GBM progression by inducing the polarization of M0 macrophages into M2 macrophages. In addition, we showed that FAP could induce the expression of CXCL8 at the protein and RNA levels in vitro. Moreover, based on the analysis of TCGA data, we found that the expression of CXCL8 was positively correlated with the expression of CD206, CD163, CD204, and CD301, markers of M2 macrophages, in both LGG and GBM (Figure S2). Meanwhile, we examined the phagocytic activity of U937‐derived macrophages after 48 h of culture with the supernatant of LN229 cells from the control group and FAP‐knockdown group, and no significant difference in phagocytic activity was found (Figure S3). One of the possible reasons is that the percentage of M2‐type macrophages is relatively low so the difference in phagocytic activity is not significant between groups with or without FAP knockdown. And another possible reason is that the assay kit is not sensitive enough to distinguish the difference in phagocytic activity of the two groups of macrophages clearly. Taken together, we considered that FAP remodels the phenotype of macrophages toward the M2 type by potentially inducing CXCL8 expression in GBM. However, it is not clear what role CXCL8 plays in this process, and the detailed regulatory mechanism remains to be elucidated in future studies.

In this study, we used a BALB/c nude mouse model subcutaneously injected with a human‐derived GBM cell line to investigate the effect of FAP on tumor invasiveness and observe the therapeutic effect of PT100 on subcutaneous tumors. The results showed that tumor invasiveness can be effectively inhibited by inhibiting FAP expression, and PT100 exerted an exciting effect on inhibiting the invasion of tumor cells. On the other hand, an orthotopic allograft model in C57BL/6 mice was used to illustrate the effect of PT100 and to determine whether PT100 could enhance the therapeutic effect of TMZ, which is commonly used in the treatment of GBM. According to the results, PT100 could enhance the efficacy of TMZ, which was shown by a smaller tumor volume and a longer survival time.

However, our study has several limitations. The current study tested only two glioblastoma cell lines (LN18 and LN229), considering the heterogeneity of glioblastoma, more glioblastoma cell lines should be tested. Additionally, although we demonstrated the effect of FAP on the process of epithelial‐mesenchymal transition and M2 macrophage polarization in glioblastoma, it is not clear what role FAP plays in those processes, and the detailed regulatory mechanism remains to be elucidated in future studies. Furthermore, metabolic reprogramming is a common feature of cancer progression and metastasis. Cancer cells rewire metabolism to sustain both their growth and to adapt to harsh microenvironments. And immune environment also plays an important role in tumorigenesis and cancer progression. More and more studies have shown that the alteration of the metabolic and immune environment may determine the efficacy of anti‐glioma treatment. And some advanced modalities have been developed allowing for the microenvironment in glioblastoma to be determined.^44^, ^45^ The impact of FAP on glioblastoma metabolic and immune microenvironment becomes achievable. Further studies could be carried out using some advanced modalities in the future.

In conclusion, we showed that upregulated FAP expression indicated a poorer prognosis in both LGG and GBM and was correlated with non‐codeletion of 1p19q, wild‐type IDH1 status, and old age. FAP promotes cell invasion and migration by activating the EMT program. FAP could induce CXCL8 expression to increase M2 macrophage polarization in GBM. PT100, an inhibitor of FAP, is a potent antitumor agent for GBM treatment in vivo. PT100 combined with TMZ had a better treatment outcome than TMZ alone in orthotopic allograft models. These results suggest that FAP could serve as a biomarker and novel therapeutic target for the treatment of GBM and that PT100 is a promising drug for the treatment of GBM.

## CONFLICT OF INTEREST

None of the authors have any conflict of interest to disclose regarding this study.

## Supporting information


**Figure S1.** FAP has no effect on the proliferation of GBM cells.
**Figure S2.** CXCL8 was positively correlated with the expression of markers of M2 macrophages, including CD163, CD206, CD204, and CD301, in both LGG and GBM based on TCGA dataset.
**Figure S3.** Examination of phagocytosis in U937‐derived macrophages using Cell Meter™ Fluorimetric Phagocytosis Assay Kit (AAT Bioquest, Cat# 21225).
**Table S1.** The primer used for Quantitative Real‐Time PCR in this study.

## Data Availability

The data that support the findings of this study are available from the corresponding author upon reasonable request.
