# Supplementary material for: Anti‐cancer effect of targeting fibroblast activation protein alpha in glioblastoma through remodeling macrophage phenotype and suppressing tumor progression
Source: CNS Neurosci Ther. 2022 Nov 15;29(3):878–92. doi: 10.1111/cns.14024 (PMC9928553; doi:10.1111/cns.14024)
Supplement: Supplementary file 1 — Figure S1. FAP has no effect on the proliferation of GBM cells. Figure S2. CXCL8 was positively correlated with the expression of markers of M2 macrophages, including CD163, CD206, CD204, and CD301, in both LGG and GBM based on TCGA dataset. Figure S3. Examination of phagocytosis in U937‐derived macrophages using Cell Meter™ Fluorimetric Phagocytosis Assay Kit (AAT Bioquest, Cat# 21225). Table S1. The primer used for Quantitative Real‐Time PCR in this study. [file CNS-29-878-s001.docx]

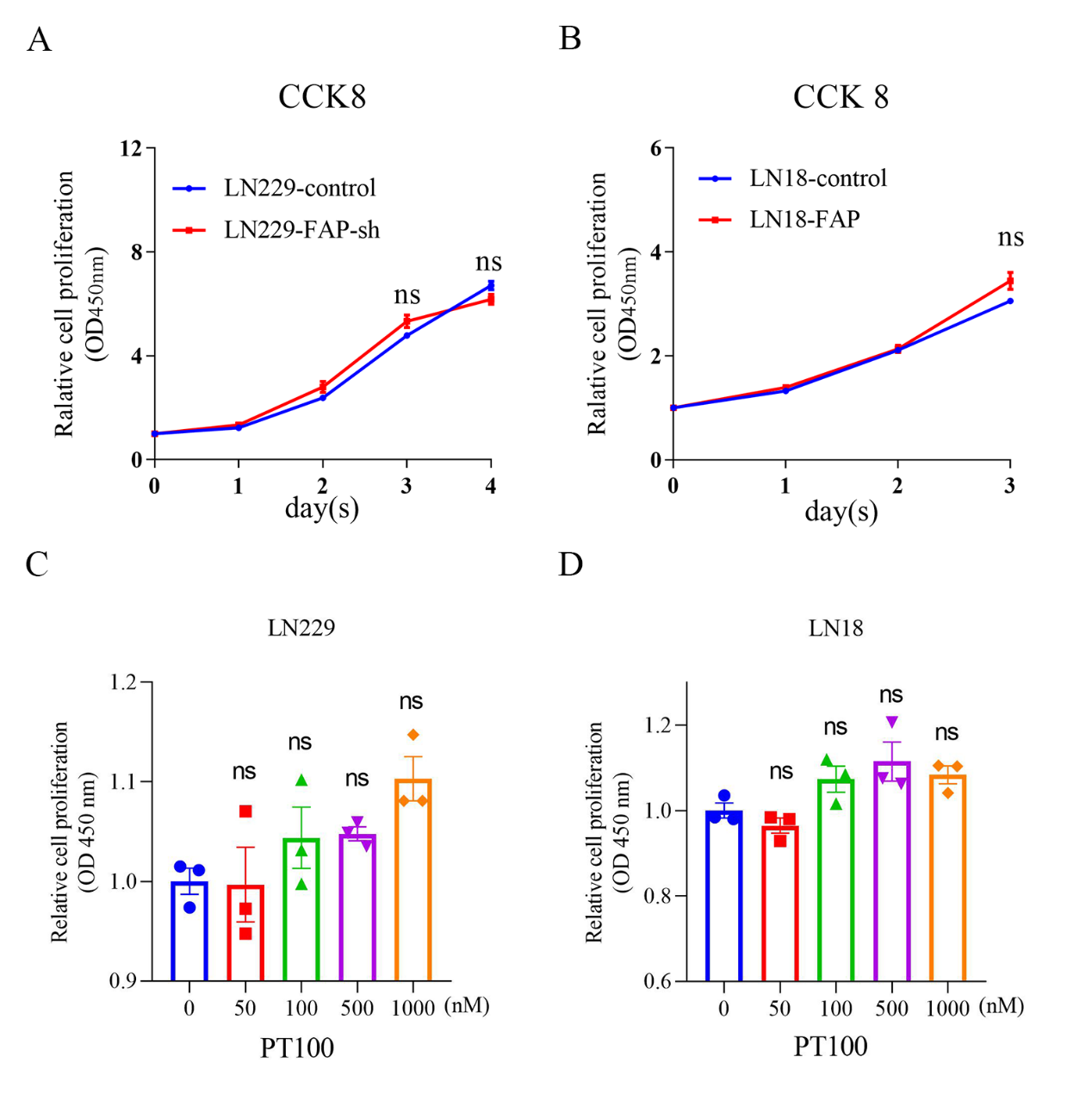


**Supplemental Figure 1:** FAP has no effect on proliferation of GBM cells. (A) The growth curve of LN229 with or without FAP knockdown. (B) The growth curve of LN18 with or without FAP overexpression. (C, D) Results of CCK-8 cell proliferation assay showed that different concentrations of PT100 had no significant effect on cell proliferation in LN229 (C) and LN18 (D) cell lines. ns: non significance.


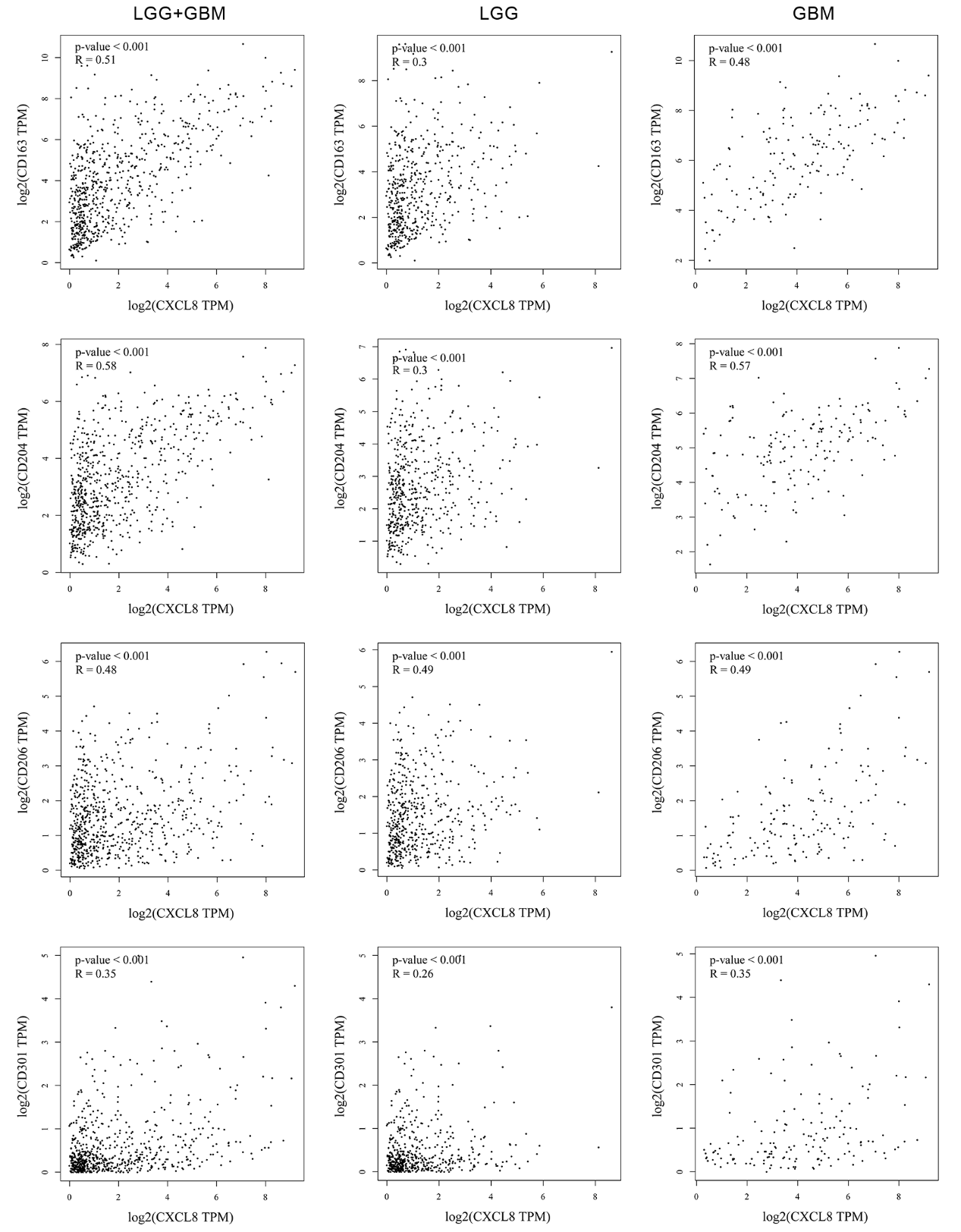


**Supplemental Figure 2:** CXCL8 was positively correlated with the expression of markers of M2 macrophages, including CD163, CD206, CD204, and CD301, in both LGG and GBM based on TCGA dataset.


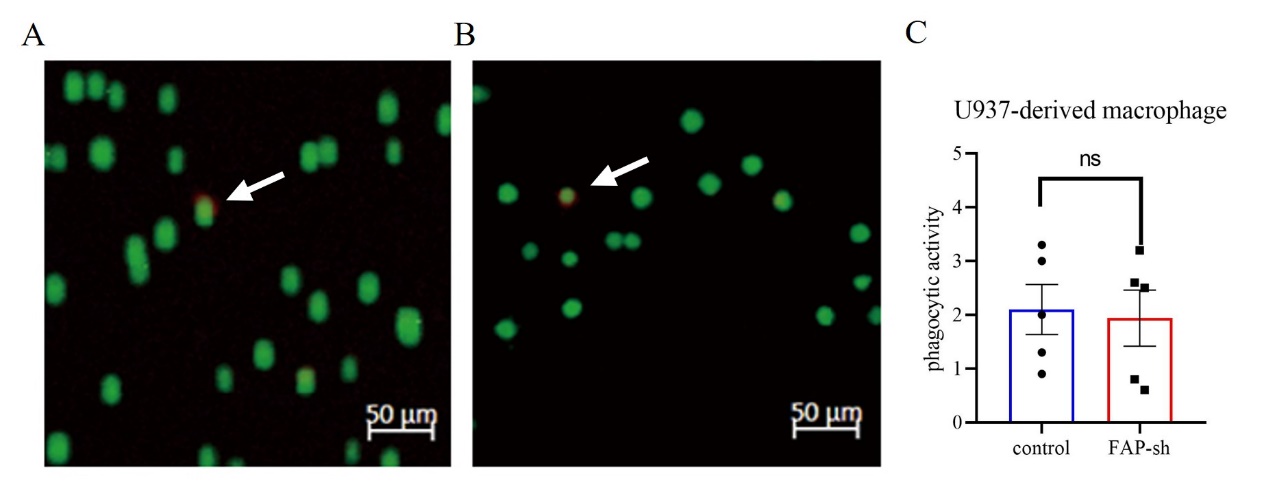


**Supplemental Figure 3:** Examination of phagocytosis in U937-derived macrophages using Cell Meter™ Fluorimetric Phagocytosis Assay Kit (AAT Bioquest, Cat# 21225). U937-derived macrophages were incubated with the conditioned medium of LN229 cells with (B) or without (A) FAP expression knocked down. There is no significant difference of phagocytic activity between the two groups (C). Green means living cells, and red means beads phagocytosed by macrophages.

**Supplementary Table 1.** The primer used for Quantitative Real-Time PCR in this study.

| name | Primer Sequence |
| --- | --- |
| FAP | forward 5′- CGATACCACTTACCCTGCGT-3′ |
|  | reverse 5′- TAACCCACGTGAGCCAACTG-3′ |
| CDH1 | forward 5′- CGAGAGCTACACGTTCACGG-3′ |
|  | reverse 5′- GGGTGTCGAGGGAAAAATAGG-3′ |
| CDH2 | forward 5′- TCAGGCGTCTGTAGAGGCTT-3′ |
|  | reverse 5′- ATGCACATCCTTCGATAAGACTG-3′ |
| TWIST1 | forward 5′- GTCCGCAGTCTTACGAGGAG -3′ |
|  | reverse 5′- GCTTGAGGGTCTGAATCTTGCT -3′ |
| SNAI1 | forward 5′- TCGGAAGCCTAACTACAGCGA-3′ |
|  | reverse 5′- AGATGAGCATTGGCAGCGAG-3′ |
| GAPDH | forward 5′- CTGCTGATGCCCCCATGTTC-3′ |
|  | reverse 5′- ACCTTGGCCAGGGGTGCTAA-3′ |
